# Healthy life expectancy and the correlates of self-rated health in Bangladesh in 1996 and 2002

**DOI:** 10.1186/s12889-015-1640-6

**Published:** 2015-03-31

**Authors:** Md Ismail Tareque, Yasuhiko Saito, Kazuo Kawahara

**Affiliations:** Department of Population Science and Human Resource Development, University of Rajshahi, Rajshahi, 6205 Bangladesh; Advanced Research Institute for the Sciences and Humanities, Nihon University, Tokyo, Japan; School of Medicine, Nihon University, Tokyo, Japan; Duke-NUS Graduate Medical School, Singapore, Singapore; Department of Health Care Management and Planning, Graduate School of Medical and Dental Science, Tokyo Medical and Dental University, Tokyo, Japan

**Keywords:** Healthy life expectancy, Self-rated health, Bangladesh

## Abstract

**Background:**

Life expectancy (LE) at birth has increased steadily in Bangladesh since its independence. When people live longer, quality of life becomes a central issue. This study examines whether healthy life expectancy (HLE) at ages 15, 25, 35, and 45 is keeping pace with LE at those ages between 1996 and 2002. It also seeks to investigate the correlates of self-rated health (SRH) in 1996 and 2002.

**Methods:**

We used data from the World Values Survey conducted in 1996 and 2002 among individuals 15 years and older. The Sullivan method was used to compute HLE. Socio-demographic differences and their association with different states of health were examined by chi-square and Pearson’s correlation tests. Multiple linear regression models were fitted to examine the correlates of SRH.

**Results:**

The results show that perceived health improved between 1996 and 2002. For males, statistically significant increases in the expected number of years lived in good SRH were found. Proportionally, in 2002, both males and females at ages 15, 25, 35 and 45 expected more life years in good health and fewer life years in fair and poor health than did their counterparts in 1996. Comparatively, males expected fewer life years spent in good health but a much larger proportion of expected life in good health than did females. Finally, in multivariate analyses, life satisfaction was the only factor found to be significantly and positively associated with SRH for males and females in both years, although in both years the association was much more pronounced for females than for males.

**Conclusion:**

This study documented changes in HLE during 1996-2002. Women outlive men, but they have a lower quality of life and are more likely to live a greater part of their remaining life in poor SRH. Life satisfaction as well as other significant factors associated with SRH should be promoted, with special attention given to women, to improve healthy life expectancy and the quality of life of the Bangladeshi people.

## Background

Bangladesh is a densely populated small country of South Asia and has made significant progress in health outcomes since independence in 1971. The decrease in both infant and child mortality and the increase in life expectancy (LE) in Bangladesh during the 20th century have been remarkable achievements. Between 1974 and 2010, LE at birth increased steadily from 53 years to 67 years for males and from 49 years to 69 years for females [1,2]. When people live longer, quality of life becomes a central issue. Increasing LE raises the question of the quality of life during these extra years: are people living longer in healthy conditions, or are people living longer in unhealthy conditions? Health expectancy is a useful tool to explore these issues.

Health expectancy, an extension of the concept of LE, is a summary measure of population health that takes into account both mortality and morbidity of a population, and it partitions the expected years of life at a particular age into healthy and unhealthy years [3]. A simple method for estimating life expectancy as a function of disability or health states was proposed by Sullivan [4] in the early 1970s. As an intuitive and meaningful summary measure combining length and quality of life, health expectancy has become a standard in the world for measuring population health [5]. Indeed, health expectancy data are invaluable for predicting future needs, evaluating health programs, identifying trends and inequalities, and planning health and social services, long term care, and pensions. Health expectancy can be computed by a variety of different health dimensions, and if self-rated health (SRH) prevalence is used in the computation, the LE in good SRH is often called healthy life expectancy (HLE). In Bangladesh, some research on the health expectancy of the elderly population 60 years old and older exists. For example, Tareque et al. [6] reported that Bangladeshi women have higher disability and shorter disability-free life expectancy than their male counterparts. In Rajshahi district of Bangladesh, it is speculated that older adults can enjoy more disability-free life expectancy by involving themselves in active aging activities [7]. A study on HLE by Tareque et al. [8] reported that in the Rajshahi district of Bangladesh, HLE declined significantly as age increased; older adults at age 60 expected about 41% of their remaining life to be in good health (LE: 15.7 years and HLE: 6.5 years), while individuals at age 80 expected only 21% of their remaining life to be in good health (LE: 4.9 years and HLE: 1.1 years). But HLE in general and levels of HLE are unknown. Therefore, using data for two dates, 1996 and 2002, this study attempts to examine whether HLE at ages 15, 25, 35, and 45 kept pace with LE at those ages in 1996 and 2002. It also seeks to investigate the correlates of SRH in 1996 and 2002.

Though LE at birth for both men and women increased between 1996 and 2002, for men, there was stagnation in LE at age 15 (56.30 years in both 1996 and 2002) and a decrease in LE at ages 25, 35, and 45 (see Table 1). However, for women, there were significant increases in LE at all ages between 1996 and 2002. In a study by Hurt et al. [9], mortality was reported to be negatively associated with socio-economic status and higher in men than in women. In particular, in Matlab, Bangladesh, age-specific mortality rates were higher in men than women after age 45. There is no published literature related to declining male LE at ages 25, 35, and 45 in Bangladesh, and it is hard to identify exactly what was driving the drop in male expectancy; it could be the effect of increased male mortality in those age groups as well as a higher incidence of traffic accidents among the male population than the female population. In a study by Mashreky et al. [10], people 18-45 years old were found to be the major victims of road traffic accidents, and more than two-thirds of road traffic accidents happened to males.

**Table 1:** **Life expectancy by age and sex in 1996 and 2002**

**Age**	**1996**		**2002**	
	**Male**	**Female**	**Male**	**Female**
0	63.96	65.38	65.59	69.70
15	56.30	57.30	56.30	59.70
25	47.10	48.10	46.70	50.30
35	37.70	38.80	37.40	40.90
45	28.90	29.80	28.40	31.40

We used the same framework for the relationship between LE and HLE and SRH proposed by a study [8] for Bangladesh to identify the direct correlates of SRH and the indirect correlates of HLE. To estimate HLE, a population measure, we used SRH prevalence obtained from survey data and mortality information obtained from published life tables. And the investigation of the correlates of SRH (used before to estimate HLE) was done using multivariate analysis. By identifying the correlates of SRH, which is used to compute HLE, we hope to contribute to the improvement of individuals’ SRH and, in turn, the improvement of their HLE.

SRH is a global measure of health assessment [11], a multidimensional concept [12], and the most unique, informative, and widely used single measure of human health status [13]. Although it is influenced by several socio-demographic and contextual factors [14], it is a simple variable that likely measures a great deal more than disease burden [15]. In spite of the variation in the wording of the question, there is extensive evidence that SRH is a potent predictor of future survival/mortality and morbidity [16,17], functional decline [18] and disability, and utilization of health care [17,19]. In addition, SRH is found to be an easy-to-use indicator of health-adjusted life expectancy in localized prostate cancer patients [20].

A significant number of studies identified several correlates of SRH in different settings. For example, the most frequently used variables and those proven to be significantly associated with SRH are age [21-24], gender [21-23,25], marital status [26], education [25], and religiosity [27,28]. After controlling for several covariates in the regression model, SRH in Bangladesh is found to be better for males than females and to deteriorate with increasing age [29].

A review study found a large proportion of published empirical data suggesting a positive association between greater religious involvement and better mental and physical health outcomes; there were relatively few studies showing no effect or a negative effect of religiosity on health outcomes [30]. Religious faith may reduce fear and provide comfort when stress occurs and, in so doing, may lend weight to the consideration of the central nervous system as a mediator of health and illness [31]. Perhaps, the nervous system represents the locus of a mechanism by which religious faith or beliefs promote well-being. Among Taiwanese older adults, higher levels of religious activity mean more expected years lived and more expected years lived without an activities-of-daily-living (ADL) disability [32]. Religiosity was found to be related to mental and physical health in the US; it predicted positive affects (affection, joy, love, happiness, contentment, caring, pride, and fondness), fewer mental health ailments, and lower levels of cognitive intrusions [33]. In addition to the possible protective effect of religiosity, there may also be a structural/social inclusion effect. Such an effect might depend on a) the average level of religiosity in the population or study sample and whether people are part of the majority or minority when they are religious or not, and b) the social composition of these two groups.

A well-established belief regarding inequalities in health around the world is that death rates and poorer SRH are higher in groups of lower socioeconomic status. In developed countries, the gradient in the association between socioeconomic status and health is well documented with individuals of higher status living longer, enjoying better health, and experiencing less disability [23,34-39]. In Poland, poor SRH was also found in older people with low education, low income, poor control over life, and chronic illness [40]. In developing countries, particularly in Bangladesh, people of lower socioeconomic status have poorer health and higher mortality [9,29] and disability [41].

Political variables such as the ruling political party (either alone or as a majority partner in a coalition), especially when in power for longer periods of time, were found to be important factors influencing a country’s income levels, the degree of social inequality, and health indicators, such as infant mortality [42]. Across the 24 Argentine provinces from 1983 to 2005, political factors were found to be positively, but weakly, associated with health outcomes [43]. Political factors affected population health positively in the advanced OECD countries [44]. Research has demonstrated that social networks are fundamental resources in the prevention of mental and physical illness. Individuals reporting to be active members of some groups, i.e. individuals with *linking social capital,* are likely to interact with other members of the groups, thereby creating networking ties [45], which help keep individuals healthy. Among Japanese people, continued social participation at advanced ages was found to be strongly associated with lower levels of mortality [46].

In a 20-year study of initially healthy men from the Finnish twin cohort, Koivumaa-Honkanen et al. [47] reported that life satisfaction (defined as interest in life, happiness, and general ease of living) was associated with decreased disease mortality after adjustment for marital status, social class, smoking, and physical activity. Perceived life satisfaction was found to be associated with SRH in Canada [48]. And in the US, it has been established as a youth developmental asset [49]. Individuals who were happier were found to have better medication adherence compared with individuals who were less happy. Among African Americans, poor medication adherence was reported to be a leading cause of excessive cardiovascular morbidity [50]. Poor life satisfaction was also found to be associated with poor SRH in several settings [51,52].

## Methods

### Data

To compute HLE for the years 1996 and 2002, two key pieces of information were needed: one, age-specific mortality information obtained from standard period life tables; and two, the proportions of the population with different states of health obtained from cross-sectional surveys for the same period as the standard period life tables.

Age- and sex-specific standard period life tables for the years 1996 and 2002 were obtained from the Human Life-Table Database [1]. Bangladesh is divided into 64 districts. The districts are further divided into sub-districts called *upazila* or *thana*. Using vital statistics collected under the Demographic Surveillance System in Matlab thana*,* nationally representative life tables were produced by the International Centre for Diarrhoeal Disease Research, Bangladesh. Nationally representative data on SRH by age for the Bangladeshi population came from the World Values Survey (WVS) waves of 1994-1998 and 1999-2004. In brief, WVS fields a standardized questionnaire among nationally representative samples of residents (sampling between 1,000 and 3,000 respondents per country). The survey covers the widest array of societies with questions on values, state of health, life satisfaction, and so on. Details on questionnaire wording, fieldwork organization, and data access can be obtained at www.worldvaluessurvey.org. In the two waves, the actual survey years for Bangladesh were 1996 and 2002, covering a roughly 6-year period. The data were collected from 1525 and 1500 individuals 15 years and older in 1996 and 2002, respectively. The response rate was 95% in 2002, and the data do not include the institutionalized population, which comprises a very small percentage of the Bangladeshi population.

### Measures

#### Outcome variable

SRH serves as the outcome measure in this study. In the 1996 WVS, SRH was assessed by a single item: ‘All in all, how would you describe your state of health these days? Would you say it is (a) very good, (b) good, (c) fair, (d) poor, or (e) very poor?’ In the 2002 WVS questionnaire, ‘very poor’ was not included in the above question. We categorized SRH into three groups: ‘good’ with a value of 3, ‘average’ with a value of 2, and ‘poor’ with a value of 1. For the year 1996, ‘very good’ and ‘good’ were grouped as ‘good’, ‘fair’ as ‘average’, and ‘poor’ and ‘very poor’ as ‘poor’. In 2002, ‘poor’ state of health alone represents the category ‘poor’, and the other responses were grouped as in 1996.

#### Independent variables

The current study includes a large set of independent variables as in previous studies e.g. [11,13,16,21,22,53] mentioned in the Background section. Four age categories (15-24, 25-34, 35-44, and 45 and above) were used for percentage distribution of respondents and their SRH to compute HLE, and a single year age was used in regression analyses. Two educational categories were created (‘illiterate’ with no formal education and ‘literate’ with incomplete primary education or higher). Two marital status categories were created (‘married’ and ‘others’), with ‘others’ including single, divorced, separated, or widowed individuals. Two religious categories were created (‘Muslim’ and ‘non-Muslim’) with ‘non-Muslim’ including Hindus, Buddhists, Christians, and others.

Religiosity was measured based on the responses to the question, ‘Independently of whether you attend religious services or not, would you say you are (a) a religious person, (b) not a religious person, or (c) an atheist?’ A respondent was considered religious when s/he said so and considered non-religious when s/he said that s/he was an atheist or not a religious person.

An individual’s class was measured based on the responses to the question, ‘People sometimes describe themselves as belonging to the working class, the middle class, or the upper or lower class. Would you describe yourself as belonging to the: (a) upper class, (b) upper middle class, (c) lower middle class, (d) working class, or (e) lower class?’ In this study, people belonging to the upper class or upper middle class were considered as upper class people, people belonging to the lower middle class as middle class people, and people belonging to the working or lower class as lower class people.

A household’s status in income scale was measured based on the response to the following question: ‘On this card is a scale of incomes on which 1 indicates the lowest income decile and 10 the highest income decile in your country. We would like to know in what group your household is. Please, specify the appropriate number, counting all wages, salaries, pensions and other incomes that come in [1 lowest decile (…) 10 highest decile].’ In our analyses, we used the above-mentioned scale variable (1-10) without any categorization.

The WVS asks people about their family’s economic situation in the past year: ‘During the past year, did your family (a) save money, (b) just get by, (c) spent some savings, and (d) spent savings and borrowed money?’ In our study, these four response categories were considered as four different categories with three dummy variables.

An individual’s financial satisfaction was measured based on the response to the question: ‘How satisfied are you with the financial situation of your household? Please use this card again to help with your answer [1 completely dissatisfied (…) 10 completely satisfied].’ Without any categorization, the above scale variable (1-10) was used in our analysis.

Control over life was measured based on responses to the question: ‘Some people feel they have completely free choice and control over their lives, while other people feel that what they do has no real effect on what happens to them. Please use this scale where 1 means no choice at all and 10 means a great deal of choice to indicate how much freedom of choice you feel you have over the way your life turns out [1 no choice at all (…) 10 a great deal of choice].’ The above-mentioned point scale was used keeping the same 1-10 point scale as originated from the WVS.

The current political situation was measured based on responses to the question: ‘How politically is this country being governed today? Again using a scale from 1 to 10, where 1 means that it is very bad and 10 means that it is very good, what position would you choose? [1 very bad (…) 10 very good].’ This point scale was also used, keeping the same 1-10 point scale as originated from the WVS.

Membership in any of seven voluntary organizations (mosque or religious organization, art or music or educational organization, labor union, political party, environmental organization, professional association, and sport or recreational organization) was used to assess respondents’ linking social capital. Because of the similarity in organizational membership in both the 1994-98 and the 1999-2004 WVS, only seven organizational membership groups were selected.

Following standard practice, the WVS measures life satisfaction by asking people how satisfied they are with their lives: ‘All things considered, how satisfied are you with your life as a whole these days? Please use this card to help with your answer [1 dissatisfied (…) 10 satisfied].’ This single-question scale has been shown to be valid for measuring life satisfaction in large-sample surveys [54,55]. It is important to note that the above-mentioned point scales were used keeping the same 1-10 point scales as originated from the WVS.

### Estimation of HLE

HLE combines the mortality and morbidity experience of a population into a single composite indicator. To compute HLE for the years 1996 and 2002, the Sullivan [4] method was used. Age-specific mortality data from published life tables were combined with age-specific proportions of life with different states of health obtained from the surveys. The proportions were used to calculate the person-years of life lived with different states of health for the age intervals of the population in the life tables. The person-years with different states of health for each age are summed up from age x onwards to the end of the table to obtain the total person-years with different states of health. Then, health expectancies in different states of health were obtained from the basic life tables by dividing total person-years by the survivors at that age. For more details on computation of HLE and confidence intervals using the Sullivan method, see Jagger et al. [56].

### Statistical analyses

Univariate analysis was performed to see the socio-demographic distribution of the study population in 1996 and 2002. Socio-demographic differences and their association with different states of health were examined by chi-square tests; correlation between continuous variables was tested using Pearson’s correlation test (results not shown). Finally, two multiple linear regression models for SRH by sex were fitted to examine the correlates of SRH in 1996 and 2002. All variables significant in chi-square and correlation tests at level p < 0.20 were included in a multiple linear regression. Because of a high correlation between income and individuals’ class (Year 1996: Males: 0.68, Females: 0.67; Year 2002: Males: 0.64, Females: 0.76), income alone was included in the multivariate analysis. Moreover, multicollinearity of the variables was checked by examining the Tolerance value (the reciprocal of the Tolerance value is the Variance Inflation Factor, VIF), which was ≥ 0.60 [57] and, consequently, financial satisfaction was excluded from the multivariate analyses. The entire analysis of the study was conducted using STATA/SE 12.1 (StataCorp LP, College Station, Texas, United States of America).

### Ethical considerations

Publicly available WVS data were used for the current study. As the de-identified data for this study came from secondary sources, this study does not require ethical approval.

## Results

As can be seen from Table 2, in both 1996 and 2002, the highest percentage of females and males reporting having good SRH occurred in the 15-24 and 25-34 age groups, respectively. Older individuals were more likely to be in poor health than their younger counterparts. The proportion of respondents in poor health increased with age for both sexes in both 1996 and 2002. Comparatively, higher percentages of individuals in 2002 than in 1996 reported having good health across all age groups. In other words, perceived health improved between 1996 and 2002.

**Table 2:** **Percentage distribution of SRH by age groups and sex in 1996 and 2002**

**Age groups**	**Male**			**Female**		
	**Good health**	**Average health**	**Poor health**	**Good health**	**Average health**	**Poor health**
	Year 2002					
15-24	61.34	33.61	5.04	68.71	27.89	3.40
25-34	64.77	31.32	3.91	60.82	36.36	2.82
35-44	57.33	38.36	4.31	43.24	50.68	6.08
45+	50.52	40.72	8.76	53.85	38.46	7.69
	Year 1996					
15-24	48.15	47.22	4.63	47.65	48.24	4.12
25-34	56.52	39.53	3.95	44.81	46.67	8.52
35-44	51.82	40.91	7.27	44.79	45.40	9.82
45+	37.22	49.25	13.53	41.33	41.33	17.33

Table 3 shows LE, HLE, and proportion of life spent in different states of SRH for 15, 25, 35, and 45 year-old Bangladeshi males in 1996 and 2002. As discussed in the background section of the current study, between 1996 and 2002, life expectancy of 25, 35, and 45 year-old men showed a decrease of about 3 to 6 months, while there was no change for 15 year-old men. There was a statistically significant increase in the expected number of years lived in good SRH for all ages; but it was not statistically significant for 45 year-old men. A decrease in fair and poor HLE was also found between 1996 and 2002. Proportionally, male individuals in 2002 expected more life years in good health and fewer life years in average and poor health than individuals in 1996.

**Table 3:** **Life expectancy and healthy life expectancy for Bangladeshi males by age in 1996 and 2002**

**Age**	**Life expectancy (years)**	**Expected number of years in:**						**Proportion in:**		
		**Good health**		**Average health**		**Poor health**		**Good health**	**Average health**	**Poor health**
		**Years**	**95% CI**	**Years**	**95% CI**	**Years**	**95% CI**	**Percent**	**Percent**	**Percent**
Year 2002										
15	56.30	31.57‡	28.51-34.64	21.05†	17.52-24.57	3.66 ns	0.00-7.99	56.08	37.39	6.49
25	46.70	25.64†	22.77-28.52	17.83 ns	14.60-21.06	3.18 ns	0.00-7.18	54.91	38.18	6.81
35	37.40	19.54†	16.69-22.38	14.98 ns	11.84-18.11	2.84 ns	0.00-6.74	52.24	40.04	7.60
45	28.40	14.34 ns	11.53-17.15	11.56 ns	8.48-14.63	2.50 ns	0.00-6.32	50.49	40.69	8.80
Year 1996										
15	56.30	25.32	22.18-28.46	25.79	22.78-28.79	5.20	1.28-9.11	44.98	45.81	9.23
25	47.10	20.86	17.98-23.74	21.44	18.71-24.16	4.81	1.29-8.33	44.29	45.51	10.22
35	37.70	15.48	12.68-18.29	17.78	15.19-20.37	4.48	1.13-7.84	41.06	47.16	11.89
45	28.90	10.73	7.99-13.48	14.23	11.76-16.70	3.90	0.67-7.12	37.14	49.23	13.48

**Notes:** CI: confidence interval; Difference from 2002 to 1996: ‡ < 0.05; † <0.10; ns: not statistically significant.

Table 4 shows LE, HLE, and proportion of life spent in different states of SRH for Bangladeshi females in 1996 and 2002. In contrast to the decrease in male LE between 1996 and 2002, female LE showed an increase of about 1.6 to 2.4 years. Also, increases in the expected number of years lived in good SRH were found across all ages. As in Table 3, a decrease in average and poor HLE was found between 1996 and 2002. Proportionally, females in 2002 expected more life years in good health and fewer life years in average and poor health than individuals in 1996, again in line with the results of Table 3. But comparing Tables 3 and 4, it can be said that males in 2002 expected fewer life years spent in good health but a greater proportion of expected life in good health than females.

**Table 4:** **Life expectancy and healthy life expectancy for Bangladeshi females by age in 1996 and 2002**

**Age**	**Life expectancy (years)**	**Expected number of years in:**						**Proportion in:**		
		**Good health**		**Average health**		**Poor health**		**Good health**	**Average health**	**Poor health**
		**Years**	**95% CI**	**Years**	**95% CI**	**Years**	**95% CI**	**Percent**	**Percent**	**Percent**
Year 2002										
15	59.70	33.26†	27.45-39.07	22.91 ns	16.18-29.63	3.53 ns	0.00-11.79	55.71	38.37	5.91
25	50.30	26.71 ns	20.91-32.51	20.35 ns	13.69-27.01	3.23 ns	0.00-11.42	53.10	40.45	6.42
35	40.90	20.92 ns	15.09-26.76	16.94 ns	10.25-23.63	2.99 ns	0.00-11.21	51.16	41.42	7.30
45	31.40	16.88 ns	11.09-22.68	12.08 ns	5.39-18.77	2.42 ns	0.00-10.61	53.77	38.48	7.70
Year 1996										
15	57.30	25.00	19.83-30.17	25.32	20.15-30.49	7.03	0.82-13.24	43.63	44.19	12.27
25	48.10	20.55	15.41-25.68	20.82	15.69-25.95	6.72	0.59-12.84	42.72	43.29	13.97
35	38.80	16.37	11.23-21.51	16.46	11.32-21.60	5.97	0.00-12.09	42.19	42.41	15.39
45	29.80	12.31	7.14-17.48	12.34	7.17-17.51	5.16	0.00-11.28	41.31	41.41	17.30

**Notes:** CI: confidence interval; Difference from 2002 to 1996: † <0.10; ns: not statistically significant.

Table 5 provides the distribution of the socio-demographic characteristics of the study population in 1996 and 2002. The average age of respondents was 35 in 1996 (34 in 2002). Compared to female respondents, a higher percentage of male respondents was sampled in both 1996 and 2002. More respondents were found illiterate in 2002 than in 1996; the average year of schooling was a bit lower in 2002 than in 1996. Compared to 1996, a higher percentage of respondents were married, Muslim, and religious in 2002; and a higher percentage of respondents came from the lower class, had linking social capital, and had good SRH. The household status in income scale was higher (4.68 vs. 4.48), but financial satisfaction was lower (5.57 vs. 6.07) in 2002 than in 1996. Comparatively, respondents felt they had less control over life, were in a worse political situation, and had lower life satisfaction in 2002 than in 1996.

**Table 5:** **Socio-demographic characteristics of the study population in 1996 and 2002**

	**1996**		**2002**	
**Variables**	**N (1525)**	**Percentage**	**N (1500)**	**Percentage**
Age groups				
15-24	278	18.23	267	17.80
25-34	523	34.30	602	40.13
35-44	383	25.11	380	25.33
45+	341	22.36	251	16.73
Sex				
Male	847	55.54	829	55.27
Female	678	44.46	671	44.73
Educational level				
Illiterate	155	10.16	190	12.67
Literate	1368	89.70	1297	86.47
Missing	2	0.13	13	0.87
Marital status				
Married	1141	74.82	1180	78.67
Others	384	25.18	320	21.33
Religion				
Non-Muslim	219	14.36	122	8.13
Muslim	1306	85.64	1378	91.87
Religiosity				
Non-religious	237	15.54	43	2.87
Religious	1240	81.31	1331	88.73
Missing	48	3.15	126	8.40
Individual’s class				
Lower	325	21.31	412	27.47
Middle	771	50.56	663	44.20
Upper	394	25.84	405	27.00
Missing	35	2.30	20	1.33
Family’s economic situation in past year				
Saved money	346	22.69	443	29.53
Got by	514	33.70	830	55.33
Spent savings	160	10.49	131	8.73
Spent savings & borrowed	147	9.64	84	5.60
Missing	358	23.48	12	0.80
Linking social capital				
No	750	49.18	529	35.27
Yes	775	50.82	971	64.73
Self-rated health				
Good health	714	46.82	874	58.27
Average health	685	44.92	552	36.80
Poor health	126	8.26	71	4.73
Missing	-	-	3	0.20
	Mean	SD	Mean	SD
Age (in years)	35.45	12.28	33.96	11.52
Years of schooling	4.82	2.16	4.70	2.64
HH status in income scale	4.48	2.10	4.68	1.83
Financial satisfaction	6.07	2.35	5.57	2.23
Control over life	6.32	2.68	5.65	2.27
Current political situation	6.84	2.14	5.32	2.39
Life satisfaction	6.41	2.25	5.78	2.18

**Notes:** SD: standard deviation; HH: household.

Table 6 presents the results of multiple linear regressions examining the correlates of SRH with adjustments for factors that were associated with SRH in chi-square and correlation tests in 1996 and 2002. Age was found to have a significant negative effect on SRH. This means older individuals were more likely to report poor SRH in 1996 and 2002, but in 1996 these results were not significant for males. In both years, educational level was found to have a significant positive effect on SRH for females only. Literate females were more likely to have good SRH than illiterate females in both the survey years. The disadvantage of religiosity for SRH was found to be significant for both the male and female population in 1996; but it became insignificant in 2002. For males in 1996, control over life was positively and significantly correlated with SRH, but was insignificant in 2002. For females in 2002, however, it was found to have a significant negative effect on SRH. In 1996, current political situation was found to have a positive and significant effect on SRH only for males. The male population reporting in 1996 that Bangladesh was politically well governed had a higher probability of reporting good health. In 1996, having linking social capital was also found to have a significant positive effect on SRH only for males. Most strikingly, life satisfaction was the only factor found to be significantly and positively associated with SRH for both males and females in both years, demonstrating that individuals with life satisfaction are more likely to have good SRH. In addition, this variable had a growing effect on SRH between 1996 and 2002, and this for females more than for males in both years.

**Table 6:** **Multiple linear regression on SRH in 1996 and 2002**

**Variables**	**Male**				**Female**			
	**B**	**95% CI**	**B**	**95% CI**	**B**	**95% CI**	**B**	**95% CI**
	**1996**		**2002**		**1996**		**2002**	
Age	ns	-	-0.008*	-0.012, -0.003	-0.070†	-0.143, 0.004	-0.006‡	-0.012, 0.000
Educational level: Illiterate (ref)								
Literate	ns	-	ns	-	0.238‡	0.027, 0.450	0.160‡	0.007, 0.314
Religiosity: Non-religious (ref)								
Religious	-0.140‡	-0.272, -0.008	ns	-	-0.283*	-0.441, -0.124	ns	-
Control over life	0.026§	0.005, 0.046	ns	-	ns	-	-0.029†	-0.058, 0.001
Current political situation	0.037§	0.012, 0.062	ns	-	ns	-	ns	-
Linking social capital: No (ref)								
Yes	0.103†	-0.006, 0.211	ns	-	ns	-	ns	-
Life satisfaction	0.022†	-0.004, 0.047	0.024†	-0.002, 0.050	0.059§	0.022, 0.096	0.077*	0.048, 0.107
Constant	1.742*	1.334, 2.149	2.456*	2.061, 2.852	1.863*	1.344, 2.382	1.958*	1.459, 2.458

**Notes:** B: regression coefficient; ref: reference category; Significance level * <0.001, § <0.01; ‡ < 0.05; † <0.10; ns: not statistically significant; All models are controlled for the effects of variables significant at 20% in chi-square and correlation tests.

## Discussion

This study has three main findings. First, between 1996 and 2002 the study found significant improvement in perceived health and increases in expected years lived in good SRH for both the male and female population despite the decrease in male LE. And gains in HLE were much greater than gains in LE. We believe that the improvement in perceived health between 1996 and 2002 significantly increased the expected years lived in good SRH. Bangladesh is now passing through the third stage of demographic transition [58] with a combination of declining fertility since the late 1970s, declining mortality since the mid-twentieth century, and increasing LE. The improvement in SRH may in part be due to the huge strides Bangladesh has made in improving its population’s health since becoming a nation after its war of independence.

While, the gains in SRH can be partially attributed to a series of effective health sector strategies and policy processes and to a strong emphasis on delivery of health and family planning services at the community and household levels, a closer look reveals a more nuanced picture. The distribution of SRH by socio-demographic characteristics (results not shown) reveals that in both 1996 and 2002 illiterate people and people from lower classes had good SRH in lower percentages than their counterparts, but younger and married people had good SRH in higher percentages than their counterparts. So, a larger proportion of illiterate people (12.67% in 2002 vs 10.16% in 1996) and people from lower classes (27.47% in 2002 vs 21.31% in 1996) may have degraded SRH (see Table 5), while a higher proportion of younger people (83.27% from 15-44 years in 2002 vs 77.64% from 15-44 years in 1996) and married people (78.67% in 2002 vs 74.82% in 1996) may have helped improve SRH between 1996 and 2002. Research has shown that in Bangladesh SRH deteriorates as age increases [8,29]. Healthy life partners are usually chosen for marriage; therefore, married individuals may be healthier than their counterparts. In addition, in 2002, the proportion of respondents with a good family economic situation in the past year was larger than in 1996, which may have helped further improve SRH between 1996 and 2002. In the current study, the observed larger prevalence of good SRH in 2002 than in 1996 may also have been the result, in part, of the two different response categories used for assessing SRH in the 1996 and 2002 WVS. People rated their health based on 5 response categories in the 1996 WVS, whereas they rated their health based on 4 response categories in the 2002 WVS. Jürges et al. [59] found that a slight change in the verbal response categories for SRH elicited different assessments for the World Health Organization and United States questionnaire versions in five European countries, because people recalibrated SRH relative to the new response categories. The five response categories used in the WHO questionnaire are very good, good, fair, poor, and very poor, whereas the five response categories used in the United States questionnaire are excellent, very good, good, fair, and poor.

Second, males in 2002 expected fewer life years spent in good SRH but a larger proportion of expected life in good SRH than females. Mortality has been reported to be higher for Bangladeshi men than women, particularly at older ages [9]. As a result of higher mortality among men than women, a decrease of 0.3-0.5 years (about 3 to 6 months) in male LE at ages 25-45 between 1996 and 2002 has been observed. There was an increase of about 1.6 to 2.4 years in female LE at ages 15-45 between 1996 and 2002. Our study supports the findings of Barford et al. [60] that even in the poorest countries women can expect to outlive men. While it is evident that females outlive males, they have a lower quality of life and are more likely to live a greater part of their remaining life in poor SRH. This finding is in line with studies [6,61,62] indicating that women live longer than men almost everywhere, but they suffer from more illnesses and disabilities throughout their lives. And women’s health disadvantages often arise from gender inequalities, which are pervasive particularly among the poor in the developing world.

Third, life satisfaction emerged as significantly and positively associated with SRH for both males and females in 1996 and 2002, in spite of the decrease in level of life satisfaction between 1996 and 2002. Life satisfaction and the other variables are thought to be associated with health in the current study, but health is associated with all these variables too. We therefore tested whether an interaction between life satisfaction, sex, religiosity, and income was significantly associated with SRH in both 1996 and 2002 and found the results not to be significant. In a study on adolescents in the USA [51], SRH was used as an explanatory variable and perceived satisfaction as an outcome, and poor SRH was significantly found to reduce life satisfaction, regardless of race or gender. In other words, reduced life satisfaction is associated with poor SRH. In a study in Australia [52], conversely, life satisfaction was used as an independent variable, and the people with more life satisfaction were found to report better health. In our study, life satisfaction was also found to be a significant correlate of SRH in both 1996 and 2002. Because life satisfaction is an indirect correlate of HLE, attention must be paid to life satisfaction in order to improve the HLE of the Bangladeshi people.

Using Chi-square and correlation tests, this study also identified factors significantly associated with SRH such as age, educational level, religiosity, individual’s class, household status, financial satisfaction, control over life, current political situation, linking social capital, and life satisfaction. Higher religiosity has been found to be associated with better SRH in several studies [27,28]. The current study, however, showed the opposite result for religiosity. This may be due to the fact that in 1996 individuals aged 45 and over, illiterate people, and people with lower control over life exhibited a comparatively greater religiosity than their counterparts. In 2002, individuals aged 45 and over, women, illiterate people, and people from a lower household status in income scale exhibited a comparatively greater religiosity than their counterparts.

### Limitations

This study has a few limitations. First, the sample size is limited, and the institutionalized population was not taken into account in the computation of HLE. If individuals living in institutions have poorer health than individuals residing in the community, not taking into account the institutionalized population could overestimate HLE, especially at older ages [3]. Here, we assume that people living in institutions exhibit the same distribution of health conditions as people in the community at large. Second, the subjective nature of SRH, rather than health assessments by physician diagnoses, may have introduced gender bias in the findings [63]. Third, the response category ‘very poor’ was absent for the question on SRH in 2002. This missing category may have induced study respondents to over-rate their health in 2002. Jürges et al. [59] report that people recalibrated SRH relative to new response categories. Fourth, there is the possibility healthy people were overrepresented in 2002. Though the response rate is very high (95%), 5% of the people could have been ill during the survey and therefore either have been unable or have refused to participate. And interviewers may have replaced sick people who could not or refused to participate in the survey with healthy people who agreed to participate. We were unable to identify the 5% of people who did not participate and assume that the impact on research findings of including them would have been minimal. Finally, due to the unavailability in the data sets of the exact income of respondents, we had to rely on the income scale.

## Conclusions

Although improvements in HLE are much greater than improvements in LE during the period 1996-2002, additional improvements are essential. This kind of research with recent data could play a very important role in identifying trends and inequalities, planning health and social services, evaluating health programs, and predicting future needs. Women outlive men, but they have a lower quality of life and are more likely to live a greater part of their remaining life in poor SRH. Thus, women should receive special attention and care, particularly at older ages. Furthermore, to improve the healthy life expectancy as well as quality of life of the Bangladeshi people, efforts should be made to promote life satisfaction and the other factors significantly associated with SRH.
